# Papillomatosis cutis lymphostatica

**DOI:** 10.1002/ccr3.671

**Published:** 2016-09-07

**Authors:** Dogu Aydin, Michael Heidenheim

**Affiliations:** ^1^ Department of DermatologyZealand University HospitalRoskildeDenmark

**Keywords:** Diabetes complications, lymphatic vessel, lymphatic vessel damage, lymphedema, papillomatosis, papillomatosis cutis

## Abstract

Papillomatosis cutis lymphostatica is a benign, usually asymptomatic and underreported condition resulting from primary lymphedema or damage of lymphatic vessels due to diabetes. Cases have only been published sporadically. The presented image may help future colleagues to establish the diagnosis.

## Quiz question

What is this condition and what is the etiology?

**Figure 1:**
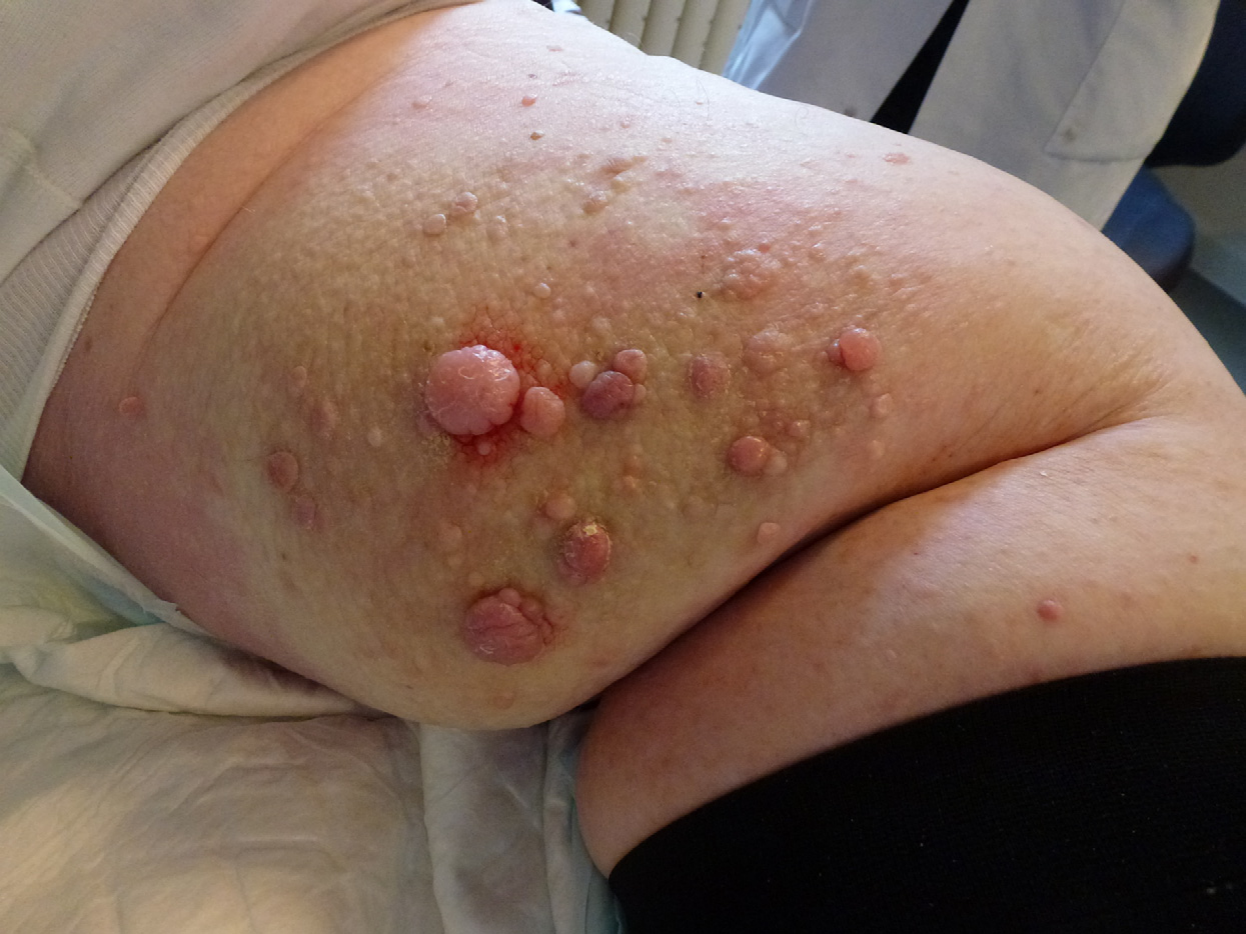
Papillomatosis cutis lymphostatica on the left thigh of a 67‐year old morbidly obese man.

A 67‐year‐old morbidly obese man (BMI 53), suffering from noninsulin dependent diabetes, presented himself with a 1‐year history of enlarged and asymptomatic papules on both popliteal areas. Physical examination revealed several confluent, partially hyperkeratotic and verrucous cobblestone‐like papules and nodules on both popliteal areas. (see figure 1). Clinical and histological findings supported the suspicion of papillomatosis cutis lymphostatica (PCL). Papillomatosis cutis lymphostatica is benign and an unusual sequela to primary lymphedema 1, 2. Papillomatosis cutis lymphostatica can also result secondary to conditions related to damage of lymphatic vessels, including diabetes. Papillomatosis cutis lymphostatica can damage skin and increase the risk of skin infections 1. Differential diagnosis includes nodular pretibial myxedema, lymphedema, etc. Treatment options are limited to compression, topical ointments, and surgery. The patient was treated successfully with compression and CO2 laser excision 1.

## Conflict of Interest

None declared.
